# Heat-Resistant Ferroelectric-Polymer Nanocomposite with High Dielectric Constant

**DOI:** 10.3390/ma11081439

**Published:** 2018-08-15

**Authors:** Nikolay Mukhin, Valentin Afanasjev, Irina Sokolova, Dmitry Chigirev, Rene Kastro, Lyudmila Rudaja, Galina Lebedeva, Aleksandr Oseev, Andrey Tumarkin

**Affiliations:** 1 Institute of Micro and Sensor Systems, Otto von Guericke University of Magdeburg, 39106 Magdeburg, Germany; 2 Faculty of Electronics, Saint Petersburg Electrotechnical University “LETI”, 197376 Saint Petersburg, Russia; vpafanasiev@mail.ru (V.A.); imsokolova@mail.ru (I.S.); dachigirev@mail.ru (D.C.); avtumarkin@yandex.ru (A.T.); 3 Department of Physical Electronics, Herzen State Pedagogical University of Russia, 191186 Saint Petersburg, Russia; recastro@mail.ru; 4 Saint Petersburg State Institute of Technology, 190013 Saint Petersburg, Russia; 9241890@mail.ru; 5 Institute of Macromolecular Compounds, Russian Academy of Sciences, 199004 Saint Petersburg, Russia; lebedeva_gk@bk.ru; 6 FEMTO-ST Institute, Univ. Bourgogne Franche-Comté, CNRS, 15B avenue des Montboucons, 25030 Besançon cedex, France; aleksandr.oseev@femto-st.fr

**Keywords:** composites, impedance spectroscopy, dielectric properties

## Abstract

The high dielectric constant ferroelectric-polymer nanocomposite was developed for producing the heat-resistant and chemical stable planar layers. According to the composite coatings formation conditions, the following value ranges of dielectric constant and loss factor were received: 30–400 for dielectric constant and 0.04–0.1 for loss tangent, accordingly. Unlike of composite components, the obtained composite material is characterized by thermo-stability of electrical parameters up to 250 °C. The dielectric frequency spectra of the composite exhibit two clearly visible peaks in contrast to the spectra of the polymer and ferroelectric ceramics. The developed composite material can be used as a built-in film capacitors material in microelectronic devices.

## 1. Introduction

A synthesis of new polymeric composites with ferroelectric fillers and study of their properties are urgent tasks of the growing practical application of these materials in various fields of technology, such as radio-, opto- and acousto-electronics, nonlinear optics, etc. [1,2,3,4,5,6]. The choice of manufacturing method of the composite (dielectric matrix/nanodispersed ferroelectric filler) would greatly eliminate uncertainty in the microstructure and the size of the ferroelectric grains. By changing the properties of the matrix, it is possible to vary characteristics and functionality of composites in a fairly broad range.

Nowadays various ferroelectric/polymer composite coatings are known. Ferroelectric polyvinylidenfluorid (PVDF) [7,8], its copolymer with hexafluoropropylene [9], as well as nonferroelectric substances such as polyimide [10], polyurethane [11], polytetrafluoroethylene [12], polyvinyl chloride [13], polybenzoxazol [14] and epoxy polymers [15,16,17] are most often used as a matrix in composite systems. Nanodisperse BaTiO_3_ [7,8,9,17,18], Pb(Zr,Ti)O_3_ (PZT) [14,15,19] and Ba(Fe_0.5_Nb_0.5_)O_3_ [20,21] are most often used as a ferroelectric filler.

The properties of the polymer nanocomposite depend on the composition of the filler and the matrix, on the filler concentration, the grain size of the nanodispersed filler [18,22], the level of aggregation of the nanodispersed filler, the nature of the interactions at the filler/matrix interface [7,17,21], the homogeneity of the distribution filler in the matrix. This creates the conditions for the possibility of controlling the properties of composites by varying these factors.

Required dielectric parameters of composites can be obtained by solving two technological problems. First, the choice of the composition of both the ferroelectric filler and the polymer matrix, as well as the technology of introducing the filler into the matrix. Secondly, the modification of the conditions on the interface between the filler and the polymer matrix.

A serious technological task is to prevent aggregation of particles and to ensure the homogeneity of the distribution of particles in the matrix. This problem can be solved by encapsulating the nanodispersed filler particles with dielectric shells. Encapsulation of filler nanoparticles is made by various substances: dopamine, silane, aluminum oxide [22,23]. In this case, a dual function of such shells is realized. First, the aggregation of nanodispersed ferroelectric filler particles is prevented and the matching of various materials such as ceramics and polymer on the interface is improved. In addition, the result of introducing a dielectric shell around the ferroelectric nanoparticles is also a significant reduction in the conductivity and dielectric losses in the composite. However, the question of the effect of interfaces on the ferroelectric properties of the filler remains unclear.

In this study, the problem of technology development for fabrication of a heat-resistant ferroelectric/polymer composite coating with high dielectric constant and electrical strength as well as reduced value of the dielectric loss tangent is discussed. There are main problems that arise during the formation of such systems: reduction of a size and aggregation degree of ferroelectric grains and a necessity to ensure a high degree loading of the composite by ferroelectric filler.

## 2. Materials and Methods 

The object of the research is organic-inorganic composite films with a ferroelectric filler of PZT ceramic powder in a polymer matrix of polybenzoxazole. The choice of ferroelectric filler is due to the unique properties of the PZT material [24,25].

Highly hydrophobic poly(benzoxazoles) [1] are obtained by heat treatment as a result of cyclodehydration of poly(o-hydroxyamides). Polybenzoxazole films have a heat resistance up to 400 °C in air and up to 450 °C in an inert atmosphere, high chemical resistance to both inorganic acids and bases, and aggressive organic solvents as well as low moisture capacity. These characteristics of poly(benzoxazoles) in combination with good film-forming properties, strong adhesion to various substrate materials and high dielectric properties predetermined their use as protective layers in various large and super-large integral circuits [14,26] as well as in humidity sensors [27].

Key technological composite coating formation aspects are:

- usage of poly(o-hydroxyamide) as the polymeric matrix, which is a film-forming material that at the same time ensures a high adhesion of formed films to various substrates;
- usage of PZT ceramics powder as a filler with the composition: Pb_0.81_Sr_0.04_Na_0.075_Bi_0.075_(Zr_0.58_Ti_0.42_)O_3_. PZT has a Curie temperature of 240 °C and the following dielectric properties at room temperature: dielectric constant is around 2200; loss tangent is 0.02;
- mixing of primary composite components at room temperature without pre-dispersion of filler particles;
- ultrasonic dispersion of the filler in composition of the polymer suspension that does not lead to degradation of the polymer but ensures the level of dispersion of 200–300 nm for the PZT ceramics powder;
- formation of composite coating on a substrate by sedimentation;
- removal of excess polymer from the surface of the deposited composite layer;
- heat treatment of the coating; as a result of consistent thermal treatment of the coating at 150 °C (30 min), 200 °C (30 min), 250 °C (30 min), 300 °C (30 min), 350 °C (30 min), a shrinkage of a layer thickness is not more than 30% and the degree of polyheterocyclisation reaches 98–99%.

**Figure 1:**
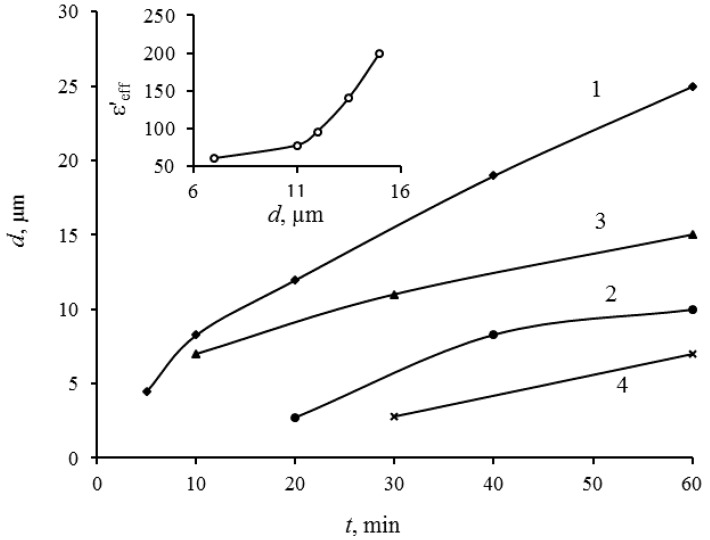
Kinetic dependences of growth of thickness of the film composite coating for various manufacturing modes. 1—∆*t* = 30 min, *n* = 6%, manual method; 2—∆*t* = 30 min, *n* = 12%, manual method; 3—∆*t* = 20 days, *n* = 6%, manual method; 4—∆*t* = 20 days, *n* = 6%, centrifugation. On the inset: dependence of effective dielectric constant of composite on the film thickness.

The films’ thickness of composites also essentially depends on such factors as viscosity of the suspension, time of decantation and method of removing an excess amount of a polymer. Figure 1 shows kinetic dependences of the composite coating thickness growth for various manufacturing modes, where *t* is the time of sedimentation. There are the following variation parameters: Δ*t*—holding time (decantation time) of the suspension after the ultrasonic treatment; *n*—concentration of the polymer solution (the content of dry polymer in grams per 100 g solution); method of removing the excess polymer layer (manual or centrifugation).

Based on the obtained dependences, an increase in the suspension viscosity (accordingly, the concentration increase of the polymer solution) significantly reduces the settling process of the filler particles. The noticeable decrease in the growth rate of the film thickness for samples obtained from the suspension that has passed decantation during 20 days in comparison with samples obtained from the freshly prepared suspension (Figure 1, curves 1,3) could be explained by the fact that during 20 days of decantation the finest fraction of the filler particles remains in suspension state. An application of the centrifugation procedure to remove excess polymer layer from the surface allows to obtain sufficiently thin films at low rotational speeds of the centrifuge (Figure 1, curve 4). Such kinetics of the composite films growth allows to expect a manageability of the manufacture process of composite coatings and reproducibility of obtained composites parameters. This is particularly important with respect to composite coatings in the thickness range of 1–3 µm.

To study dielectric properties of composite films and its components separately, capacitor structures were created on their basis. The bottom electrode was fabricated as a platinum film deposited on a silicon substrate before the deposition process of composite and polymer. The top platinum electrode was formed by ion-plasma sputtering using a shadow mask with an area of 7.5 mm^2^. As a reference, a ferroelectric sample was fabricated on a basis of a PZT ceramic plate of 200 µm thickness with sputtered on both sides platinum electrodes. The thickness of the polymer coating after heat treatment was 8.1 µm. Composite coating thickness varied in the range of 2–20 µm and the effective dielectric constant of the composite varied between 30–400 respectively; accordingly, specific capacitance varied in the range of 7000–25,000 pF/cm^2^.

The measurement of dielectric parameters was carried out by “Novocontrol Concept 41” spectrometer. Measured impedance values of structures Z(f)=Re(Z)+jIm(Z) were used to calculate the dielectric constant of materials $\varepsilon^{\prime }=Im\left(Z\right)/\left(C_{0}\cdot 2\pi f{\left|Z\right|}^{2}\right)$ and loss angle tangent tgδ=−Re(Z)/Im(Z), where *f* is the frequency; $C_{0}=\varepsilon_{0}S/d$; $\varepsilon_{0}$ is dielectric constant of vacuum; *S* is the area of electrodes; *d* is the thickness of the capacitor structure.

## 3. Results

The properties of composites are largely determined by the nature of the distribution of the filler in the polymer matrix and the degree of the filler dispersion as well as by the interface interaction. Generally, the higher the degree of dispersion and the stronger the intermolecular interaction at the interface, the greater influence has the filler on the properties of the polymer composite. On the other hand, with a change in the degree of dispersion of the ferroelectric fillers, a certain change in their own dielectric properties is observed.

The dependence of the effective dielectric constant $\left({\varepsilon^{\prime }}_{eff}\right)$ of the PZT/polymer composite on the ferroelectric filler fraction (φ) can be described by several models with different degrees of approximation.

Model 1. Model of mechanical mixture. This model consists of the linear calculation of the filler fraction contained in the composite:

(1) $${\varepsilon^{\prime }}_{eff}={\varepsilon^{\prime }}_{p}\left(\varphi -1\right)+{\varepsilon^{\prime }}_{f}\varphi ,$$

where ${\varepsilon^{\prime }}_{p}$ and ${\varepsilon^{\prime }}_{f}$ are the effective dielectric constant of the polymer and ferroelectric phases, respectively. In accordance with Equation (1), the effective dielectric constant of the composite increases sharply even with a small volume content of the ferroelectric phase. However, the experiments and theoretical study show inadequacy of this model [28,29].

Model 2. Maxwell–Garnett model [30]. The model is based on an analysis of the average field values for a single spherical inclusion in a continuous medium of a polymer matrix. The corresponding expression for the effective dielectric constant is:

(2) $${\varepsilon^{\prime }}_{eff}={\varepsilon^{\prime }}_{p}\left[{\varepsilon^{\prime }}_{f}+2{\varepsilon^{\prime }}_{p}-2\varphi \left({\varepsilon^{\prime }}_{p}-{\varepsilon^{\prime }}_{f}\right)\right]/\left[{\varepsilon^{\prime }}_{f}+2{\varepsilon^{\prime }}_{p}+\varphi \left({\varepsilon^{\prime }}_{p}-{\varepsilon^{\prime }}_{f}\right)\right].$$

Equation (2) is valid for a highly dilute composite, where the ferroelectric phase forms insulated inclusions, which volume fraction is very small.

Model 3. The Bruggeman model [30] is based on the mean field theory and considers the composite in the form of repeating elements consisting of a matrix phase containing the inclusion of a spherical filler in the center. The expression for the effective dielectric constant in this case is:

(3) $$\left(1-\varphi \right)\left[\left({\varepsilon^{\prime }}_{p}-{\varepsilon^{\prime }}_{eff}\right)/\left({\varepsilon^{\prime }}_{p}+2{\varepsilon^{\prime }}_{eff}\right)\right]+\varphi \left[\left({\varepsilon^{\prime }}_{f}-{\varepsilon^{\prime }}_{eff}\right)/\left({\varepsilon^{\prime }}_{f}+2{\varepsilon^{\prime }}_{eff}\right)\right]=0.$$

The condition for the applicability of Equation (3) is the restriction on the filling factors because of the contact need between the inclusions.

Equation (3) is the most accurate with the experiment. The PZT/polymer composite samples have effective dielectric constant values in the range of 80–400, the value of PZT fraction in accordance with the Equation (3) was from 30 to 45%.

The dependence of effective dielectric constant of composite films from the film thickness for coverings thicknesses is shown on the inset of Figure 1. The coverings were obtained from the suspensions, which were under ultrasonic treatment and 20-days decantation. The growth of the effective dielectric constant in dependence of thickness can be explained by two factors: the increase of the filler loading degree of the composite with the growth of the thickness; the reduction of relative contribution of the dielectric parameters of excess polymer layer on the surface of the deposited composite.

**Figure 2:**
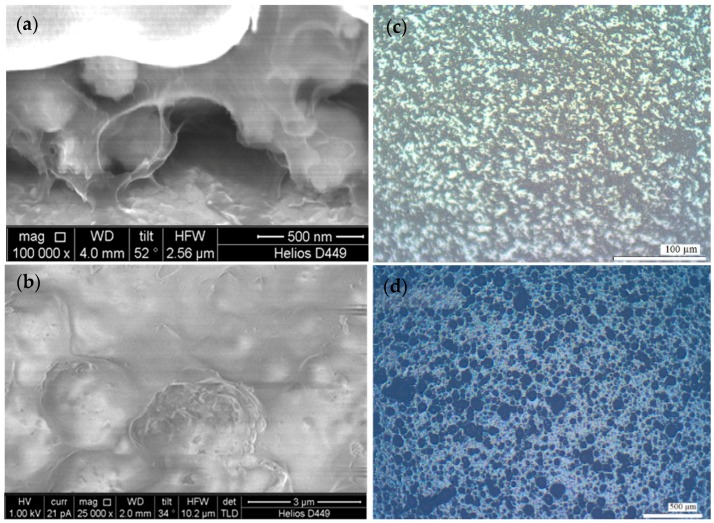
SEM image of section (**a**) and surface (**b**) of composite film and microscope-images of the composite surface: sample was obtained after ultrasonic treatment and decantation time of 20 days (**c**); sample was obtained from a suspension that was not subjected to ultrasonic treatment (**d**).

Figure 2a,b shows SEM (scanning electron microscopy) images of the obtained composite structure that indicates a high degree of filling of polymer composite by a PZT filler. Moreover, grain size of filler aggregates is about 200–300 nm, grains are uniformly distributed in the bulk of the composite, and the polymer envelops the filler grains and forms a continuous surface layer. Figure 2c,d show a comparison of the optical images of the surface of the composite samples after the heat treatment procedure. In the first case (Figure 2c) a layer of precipitated filler was obtained after ultrasonic treatment of the suspension and following decantation during 20 days. The excess amount of the polymer was removed by centrifugation. In the second case (Figure 2d), the sample was obtained from the freshly prepared suspension that was not subjected to ultrasonic treatment. The excess amount of the polymer was removed manually.

Detailed analysis of the figures reveals that the distribution of particles in the layer is not distorted under the influence of centrifugal forces. The application of the centrifugation at low speeds of rotation (500–1000 rpm) to remove excess amounts of the polymer does not substantially affect the layer of the deposited filler, and the degree of loading is not deteriorated. Since the structure of the composite coating is not violated, the centrifugation procedure can be recommended for usage. It should also be mentioned that the usage of centrifugation allows obtaining thinner films of the composite (2–3 times thinner) while maintaining other technological parameters. Partially aggregated state of the filler is caused by 20-days storage of the suspension after ultrasonic treatment. However, the average size of the particle aggregates of the filler is less than 5 µm. This is substantially lower than for the sample obtained from the suspension that was not subjected to ultrasonic treatment.

Measurements of real $\left(\varepsilon^{\prime }\right)$ and imaginary $\left(\varepsilon^{\prime \prime }\right)$ parts of dielectric constant in a broad temperature and frequency ranges were done.

**Figure 3:**
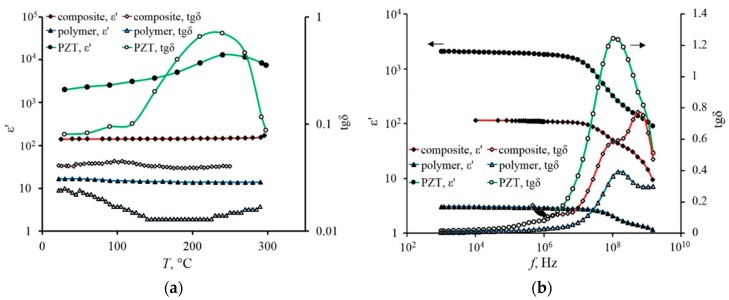
The temperature (**a**) and the frequency (**b**) dependences of ε′ and tgδ for PZT/polybenzoxazole composite (sample thickness is 13.5 µm; volume fraction of the PZT filler is about 35%) and its components: polymer (polybenzoxazole) and PZT ceramic.

Figure 3a shows a comparison of temperature dependences of the real part of dielectric constant and the dielectric loss tangent $\left(tg\delta =\varepsilon^{\prime \prime }/\varepsilon^{\prime }\right)$ that were obtained for film samples of the composite (PZT/polybenzoxazole) and its components of the polymer (polybenzoxazole) and ferroelectric ceramics (PZT). Figure 3b shows frequency dependences of the dielectric constant and dielectric loss tangent that were obtained for the composite (PZT/polybenzoxazole) and its components: polymer and PZT ceramics. The measurements were carried out at room temperature.

## 4. Discussion

The analysis of the dependencies indicates that the obtained film samples of the composite are characterized by stability of dielectric constant in the frequency range from 10 kHz to 10 MHz. In the range of 10^7^–10^9^ Hz, a broad reduction area of the dielectric constant was observed for all three materials. The composite films show also the stability of ε′ within the temperature range of 20–250 °C.

The analysis of ε′, ε″ and tgδ frequency dependencies was carried out on the basis of the following equations:

(4) $$\varepsilon \left(\omega \right)=\varepsilon^{\prime }\left(\omega \right)+j\varepsilon^{\prime \prime }\left(\omega \right);\;tg\delta \left(\omega \right)=\varepsilon^{\prime \prime }\left(\omega \right)/\varepsilon^{\prime }\left(\omega \right),$$

where ω is the angular frequency (ω=2πf). The imaginary component of the dielectric constant was described as follows:

(5) $$\varepsilon^{\prime \prime }\left(\omega \right)=-\frac{1}{\varepsilon_{0}\rho \omega }+\sum_{k}A_{k}\frac{1}{\sigma_{k}\sqrt{2\pi }}exp\left[-\frac{{\left(\omega -\omega_{k}\right)}^{2}}{2\sigma_{k}{}^{2}}\right],$$

where the first term describes the conductivity losses (ρ is electrical resistivity), and each *k*-th relaxation process is modeled using the normal distribution law and has its own relaxation time $\left(\tau_{k}=2\pi /\omega_{k}\right)$, dispersion $\left(\sigma_{k}\right)$, and amplitude $\left(A_{k}\right)$.

The real part of the dielectric constant was determined on the basis of the chosen model for epsilon using the Kramers-Kronig relation:

(6) $$\varepsilon^{\prime }\left(\omega \right)=1+\frac{2}{\pi }P\int_{0}^{\infty }\frac{\varepsilon^{\prime \prime }\left(\xi \right)\xi }{\xi^{2}-\omega^{2}}d\xi .$$

The analysis of frequency dependencies shows that two peaks are clearly visible for the composite in contrast to the spectra of the polymer and ceramics. These peaks reflect the presence of two relaxation processes in the high-frequency region. A similar coincidence was observed by authors [31] for BaTiO_3_ composite systems in polyvinyl chloride matrix. Such coincidence may indicate the presence of a significant interaction at the interface between the polymer matrix and the ferroelectric filler. Tangent spectra for all types of investigated materials (composite, ceramic, polymer) are significantly different in the magnitudes of losses and positions of the maxima. The nature of these two maxima in the dielectric spectra of the composite is required to be determined. However, as a hypothesis, the appearance of two peaks can be attributed to differences in parameters of relaxation processes that are located in the volume of ferroelectric grain and on its periphery. The relaxation times for these processes are $\tau_{1}=2.1\cdot 10^{-8}\;s$ and $\tau_{2}=1.4\cdot 10^{-9}\;s$ at room temperature. The first relaxation process has a weak activation character: $\tau_{1}\left(T\right)=\tau_{10}exp\left(-E_{1}/k_{B}T\right)$, where *T* is temperature; $k_{B}$ is Boltzmann constant; $\tau_{10}=6\cdot 10^{-8}\;s$; $E_{1}=0.027\;eV$. Parameter $\tau_{2}$ is almost independent of temperature in the investigated temperature range.

**Figure 4:**
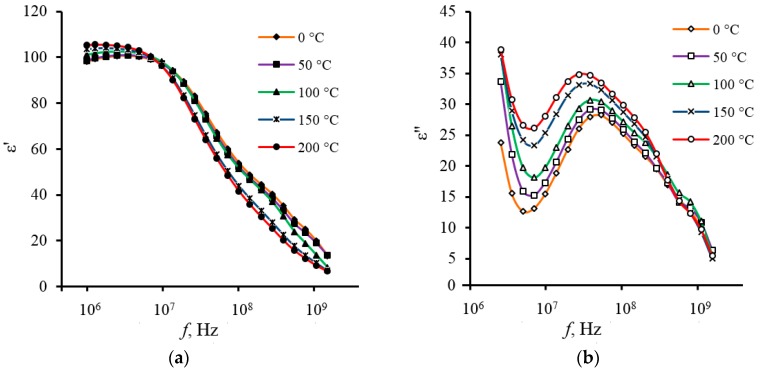
The frequency dependences of the real (**a**) and imaginary (**b**) parts of dielectric constant of PZT/polybenzoxazole composite (sample thickness is 12 µm; volume fraction of the PZT filler is about 35%) measured at different temperatures.

The important practical conclusion is the ε′ stability of composite capacitor structures in the temperature range from 0 to 200 °C, which is demonstrated in Figure 3a and Figure 4. Data were obtained in the frequency range from 1 MHz to 1.5 GHz. As the Figure 3a shows, in contrast to bulky ferroelectric the composite samples exhibit a good temperature stability of ε′ at virtually any frequency.

## 5. Conclusions

The fabrication technology of heat-resistant films of a PZT/polybenzoxazole composite system was developed, and their characteristics were studied. The basic parameters of the dispersion process of the ferroelectric powder were established: strength of ultrasonic signal, choice of dispersing medium, selection of the powder mass and process time. The minimum values of dispersion of the powder particles amounted to 200−300 nm. Correlation patterns were found between the time of deposition and the degree of the composite loading, between the viscosity of the suspension and deposition rate of filler particles, and between the time of decantation and the degree of agglomeration of the filler particles.

Depending on the combination of used technological factors, the developed technology provides the possibility of obtaining PZT/polybenzoxazole composite films with the following parameters: grain size from 200 nm to 1 µm; coating thickness from 2 to 20 µm; effective dielectric constant of the composite from 30 to 400; specific capacity from 7000 to 25,000 pF/cm^2^.

Dielectric parameters of the films, both the composite and its components have been investigated in the frequency range of 1 kHz–1.5 GHz and temperature range of 0−200 °C. The high stability of dielectric properties of the composite films was found in the frequency range from 1 kHz to 10 MHz as well as at a temperature range from 0 to 200 °C for the measured frequency range.

The obtained composite material can be used to create built-in film capacitors in microelectronic integrated devices.
